# Antimicrobial PLA-Based Composite Gels with Improved Functional Properties for Food Packaging

**DOI:** 10.3390/gels12030194

**Published:** 2026-02-26

**Authors:** Ioan Sarosi, Gertrud Alexandra Paltinean, Andrei Moldovan, Stanca Cuc, Rahela Carpa, Codruta Sarosi, Rami Doukeh, Ancuta-Elena Tiuc, Ovidiu Nemes

**Affiliations:** 1 Department Environmental Engineering and Sustainable Development Entrepreneurship, Technical University of Cluj-Napoca, 400641 Cluj-Napoca, Romania; ionut_sarosi@yahoo.com (I.S.); andrei_moldovan@mail.com (A.M.); ancuta.tiuc@imadd.utcluj.ro (A.-E.T.); 2 Department of Polymer Composites, Raluca Ripan Institute for Research in Chemistry, Babes-Bolyai University, 30 Fantanele St., 400294 Cluj-Napoca, Romania; gertrud.paltinean@ubbcluj.ro (G.A.P.); stanca.boboia@ubbcluj.ro (S.C.); 3 Department of Molecular Biology and Biotechnology, Faculty of Biology and Geology, Babes-Bolyai University, 1 M. Kogalniceanu Street, 400084 Cluj-Napoca, Romania; rahela.carpa@ubbcluj.ro; 4 Faculty of Petroleum Refining and Petrochemistry, Petroleum-Gas University of Ploiesti, 39 Bucharest Blvd., 100680 Ploiesti, Romania; rami.doukeh@yahoo.com; 5 Faculty of Chemistry and Chemical Engineering, Babes-Bolyai University, 11 Arany Janos St., 400028 Cluj-Napoca, Romania

**Keywords:** PLA, antimicrobial, functional fillers, food packaging, SEM

## Abstract

Biodegradable polymeric materials with antimicrobial functionality are increasingly explored as sustainable alternatives for food packaging. This study developed multifunctional PLA-based composite films containing controlled concentrations of active agents and evaluated their structural, mechanical, thermal, and antimicrobial properties. Five formulations were prepared: a reference PLA/glycerol diacetate blend (85/15 wt. %) and four composites with 0.5 wt. % functional fillers—grape pomace, silver–graphene oxide (GO-Ag), titanium dioxide–graphene oxide (GO-TiO_2_), or graphene oxide (GO)—with PLA adjusted to 84.5 wt. %. The films were characterized for antimicrobial activity, tensile strength, hardness (Vickers test), morphology (SEM), and thermal behavior (DSC). Mechanical testing revealed statistically significant differences (*p* < 0.05), with Vickers hardness increasing from neat PLA (13.77) to 0.5% grape pomace (16.30) and nanofiller composites (GO–Ag 18.59, GO 19.56, GO–TiO_2_ 22.7), demonstrating enhanced stiffness and efficient load transfer. Incorporation of Ag and TiO_2_ shifted endothermic transitions to higher temperatures, particularly in PLA-GT (~140 °C), indicating improved thermal stability, while neat PLA and PLA-GP showed multiple or intermediate transitions (86–92 °C). Antibacterial performance was strongly influenced by composition and surface characteristics, with PLA-GA, PLA-GT, and PLA-GO showing the greatest efficacy. These findings demonstrate that bioactive and nanostructured fillers can effectively enhance the mechanical, thermal, and antimicrobial properties of PLA, highlighting their potential for sustainable, functional food packaging applications.

## 1. Introduction

Food packaging, such as transparent foils, has become essential in the food industry due to growing consumer demands for safety and quality. Packaging that is visually appealing, colorful, and detailed tends to attract more buyers, while also aiding consumers in evaluating the product before purchase [1]. However, visual appeal alone is insufficient; the choice of packaging material is equally critical. Designing packaging that meets market demands while emphasizing biodegradability, reducing waste and environmental pollution, and minimizing health risks such as toxicity and microbial contamination represents an innovative challenge in the field.

Various sectors of the food industry—including bakery, butchery, fruits and vegetables, dairy, and beverages—have encouraged the development of biodegradable packaging solutions, with glass, paper, aluminum foil, and plastics among the most commonly used materials [2]. Although food foils are already commercially available, research on their optimal composition continues. Effective packaging must maintain freshness and protect the product from external, biological, and chemical factors [3]. Damage during handling and transportation, though sometimes minor, can facilitate bacterial contamination, underscoring the importance of careful material selection. Food foils’ constituent materials may interact with the product through chemical migration, potentially affecting quality and safety [3,4].

In conventional synthetic polymer films (e.g., polyethylene or polypropylene), protective properties are often improved by applying inorganic coatings such as metal oxides. Although these coatings enhance oxygen and moisture barrier properties, they may undergo structural and chemical changes during real food processing conditions (thermal treatment, UV exposure, acidic or fatty foods), which can promote additive migration or deterioration of the polymer matrix. In contrast, bio-based films degrade mainly through hydrolytic and microbial mechanisms, reducing long-term environmental persistence, but their stability and performance under practical storage conditions require further investigation [5].

Ecological alternatives to conventional plastics must be compatible with different food types, prevent migration of harmful substances, regulate moisture, oxygen, and light exposure, remain stable under temperature fluctuations, and avoid oxidative degradation [6]. One promising material is polylactic acid (PLA), a biopolymer derived from fermenting renewable resources such as corn, sugarcane, beet, and rice [7,8]. Recognized as safe by the US Food and Drug Administration, PLA is used in both the agri-food sector (disposable cutlery, lunch boxes, transparent containers, biodegradable waste bags, greenhouse films) [9] and the biomedical field (sutures, implants, tissue engineering scaffolds) [10]. PLA is valued as a biodegradable alternative to traditional plastics due to its transparency, high gloss, low cost, non-toxicity, and food-preserving properties [11,12].

Compared to other biodegradable polymers such as polyhydroxybutyrate (PHB), polybutylene succinate (PBS), or polycaprolactone (PCL), PLA presents a significantly lower production cost due to large-scale fermentation processes from renewable resources (corn or sugarcane). The typical market price of PLA ranges approximately between 2–4 USD/kg, whereas PHB and PHA materials may exceed 5–8 USD/kg, and PBS or PCL typically range between 4 and 7 USD/kg. Consequently, PLA represents one of the most economically feasible biodegradable polymers for food-packaging applications, balancing sustainability and industrial scalability [13].

Despite these advantages, PLA has limitations, including brittleness, low rigidity, high vapor permeability, and poor thermal stability. To overcome these drawbacks, researchers have incorporated additives and antimicrobial agents such as TiO_2_ nanoparticles, silver nanoparticles, SiO_2_, grape pomace, and graphene oxide [14,15,16,17]. The formation of PLA-based composites enhances mechanical properties and introduces antimicrobial functionalities [18,19,20,21]. Given global food safety concerns, European Union regulations require that packaging materials do not chemically alter the taste, smell, appearance, or texture of food [22]. In this context, antimicrobial agents help prevent microbial contamination, reducing the need for synthetic preservatives [17].

Titanium dioxide (TiO_2_) is a low-cost, biocompatible, non-toxic material widely used as a filler in active food packaging films. TiO_2_ enhances tensile strength, Young’s modulus, gas barrier properties, opacity, antibacterial activity, and reduces water vapor permeability when integrated with PLA [11,18,23,24,25,26,27]. Similarly, silver nanoparticles improve PLA’s mechanical strength, barrier properties against oxygen and moisture, and antibacterial effectiveness, thereby extending the shelf life of packaged products like strawberries [28,29]. According to EFSA and FDA regulations, TiO_2_ may be used in food-contact materials provided that migration remains within established safety limits. Current risk assessments indicate that migration levels below 10 mg/dm^2^ of packaging surface are acceptable [18,30,31]. In the present study, the TiO_2_ concentration was restricted to 0.5 wt. % in the polymer matrix, well below levels associated with toxicological concern. At this loading, nanoparticles remain embedded within the PLA structure, minimizing migration and supporting safe application in antimicrobial packaging systems.

Grape pomace, a byproduct of the wine industry consisting of grape skins, seeds, and stems, is rich in polyphenols, flavonoids, lipids, proteins, fibers, and minerals. Its antioxidant, antimicrobial, and non-toxic nature make it suitable for biodegradable food packaging applications, including chitosan, guar gum, and polypropylene films enriched with grape pomace extracts [28,32,33,34].

Graphene oxide, containing carbon and oxygen functional groups, has also attracted attention for food packaging applications. Incorporating graphene oxide into PLA improves tensile strength, flexibility, Young’s modulus, metal ion adsorption, and overall dispersibility compared to PLA alone [35,36].

Although numerous studies report physicochemical characterization of PLA composites, fewer investigations address their behavior under realistic storage and processing environments where moisture, UV radiation, mechanical stress, and food composition may influence migration and antimicrobial efficiency. Bridging this gap is essential for translating laboratory materials into practical packaging solutions [37,38].

The continuous evolution of food packaging highlights the need to balance environmental sustainability, food safety, and aesthetic appeal. Smart, eco-friendly packaging must meet regulatory standards, extend product shelf life, reduce waste, maintain physicochemical properties, and ensure product freshness [39].

The objective of the present study is to develop multifunctional PLA-based composite gels containing controlled concentrations of active agents and to evaluate their structural, mechanical, thermal characteristics by DSC, and antimicrobial properties in relation to real food packaging requirements. The novelty of this research lies in correlating material composition with safety constraints and potential real-life applicability rather than focusing solely on material characterization. The developed materials aim to contribute to reducing plastic pollution, limiting additive migration, and decreasing reliance on synthetic preservatives in food systems.

## 2. Results and Discussion

### 2.1. Mechanical Properties

The prepared PLA-based samples were evaluated for their mechanical performance, specifically tensile strength, elastic modulus, elongation at break, and maximum load, as summarized in Table 1.

Pure PLA exhibited a tensile strength of 1.48 MPa, an elastic modulus of 2.87 MPa, elongation at break of 180.7 mm, and a maximum load of 36.33 N. Incorporation of fillers significantly improved the mechanical properties of PLA composite gels. The PLA/0.5% grape pomace material displayed the highest performance, with tensile strength of 7.38 MPa, maximum load of 189.3 N, and an elastic modulus of 105.12 MPa, indicating increased stiffness relative to the inherently brittle PLA. However, elongation at break decreased to 66.71 mm. A similar behavior was observed for the PLA/0.5% graphene oxide and silver material.

Conversely, the addition of 0.5% graphene oxide (GO) and TiO_2_ increased the elongation at break to 248.44 mm while slightly enhancing tensile strength, attributed to the crystalline structure formed by uniform dispersion of GO and TiO_2_ nanoparticles. The elastic modulus decreased marginally to 2.60 MPa, and the maximum load reduced slightly to 35.99 N, likely due to weakened interactions between PLA and GO. Previous studies indicate that increasing the concentrations of GO and TiO_2_ further enhances tensile strength, as reported for PLA/1%GO/1%TiO_2_ composite gels [40].

Reinforcing PLA with 0.5% GO alone resulted in a significant increase in elastic modulus (102.38 MPa), a moderate increase in tensile strength (1.811 MPa), and a notable restriction in elongation at break (1588 mm), consistent with literature observations [41]. The improvements in tensile strength are attributed to the high specific surface area of GO and enhanced load transfer with the polymer matrix. Reductions in elongation at break are likely caused by agglomerated or poorly adhered filler particles, which hinder polymer chain mobility [42]. These results highlight the importance of optimizing filler content and dispersion to achieve desired mechanical properties, as compatibility between PLA and fillers depends on processing methods, formulation, and application.

Reinforcing the polymer with 0.5% graphene oxide contributes to a significant increase in the modulus of elasticity (102.38 MPa), restricts the elongation at break (1588 mm), and leads to a slight increase in tensile strength (1.811 MPa). These results are consistent with data from the specialized literature. As the percentage of graphene oxide added to the polymer mass increases, particle agglomeration occurs, which leads to a decrease in the tensile strength but an increase in the modulus of elasticity [41].

The increase in tensile strength may be due to the large specific surface area of the filler material and increased charge transfer with the polymer matrix. The decrease in elongation at break may be due to undispersed and non-adherent particles from the filler that prevent the stretching of the polymer chains [42]. Careful analysis and testing are necessary because the compatibility of filler materials with PLA may vary depending on the processing method, applicability, and desired formulation.

### 2.2. SEM Images

**Figure 1:**
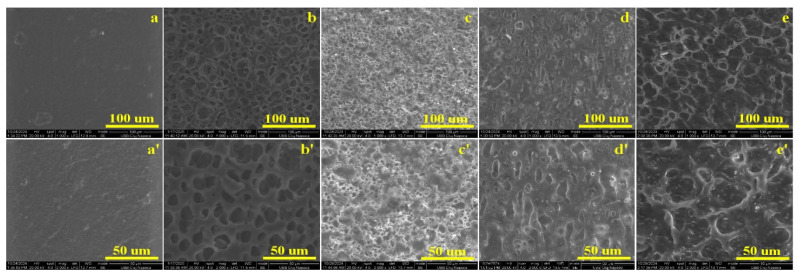
The SEM images of PLA- based composite films: (**a**) PLA; (**a′**) PLA at a magnification ×2000; (**b**) PLA-GP; (**b′**) PLA-GP at a magnification ×2000; (**c**) PLA-GA; (**c′**) PLA-GA at a magnification 2000×; (**d**) PLA-GT; (**d′**) PLA-GT at a magnification ×2000; (**e**) PLA-GO; (**e′**) PLA-GO at a magnification ×2000.

The morphology of pure PLA film and PLA-based composite films containing different nanoparticles as additives was examined using scanning electron microscopy (SEM) at magnifications of 1000× and 2000× (Figure 1).

SEM images of pure PLA (Figure 1a,a’) reveal a compact, relatively smooth surface with minor inclusions and small cracks, likely arising from the film preparation process. These features are consistent with literature reports, which note that PLA exhibits smooth fracture surfaces due to its brittle behavior at low temperatures [43]. Such surface morphology is typical for solvent-cast or melt-processed PLA films, where internal stresses and slight solvent evaporation gradients can induce microcracks.

The incorporation of fillers induced notable structural changes. PLA/0.5% grape pomace (Figure 1b,b′) displayed a porous surface with voids ranging from 5 to 50 µm. At higher magnification, two void categories were observed: (1) isolated, well-defined voids (3–20 µm) and (2) interconnected pores, indicating a heterogeneous topography. Similar effects have been reported for natural fiber or agro-waste reinforced PLA composites, where filler incorporation increases porosity and roughness due to limited polymer–filler adhesion and heterogeneous dispersion [44].

The PLA/0.5% GO-Ag composite (Figure 1c,c′) exhibited a porous, layered surface with uniformly dispersed silver nanoparticles. High-magnification images revealed networks of interconnected and non-uniform pores (1–10 µm) connected by branched structures. Literature reports suggest that strong interactions between graphene oxide and silver nanoparticles influence both the structural properties of the composite. Such interactions can enhance nanoparticle dispersion, reduce agglomeration, and create microstructural networks that improve mechanical and antimicrobial performance [45,46].

For PLA/0.5% GO-TiO_2_, SEM images (Figure 1d,d′) showed a smooth surface with overlapping graphene oxide sheets and randomly dispersed TiO_2_ nanoparticles, without evident agglomeration. The preparation process likely promotes strong interactions between TiO_2_ and GO, with TiO_2_ particles partially coated by GO sheets.

The PLA/GO composite displayed layered networks with pores ranging from 5–25 µm (Figure 1e). At higher magnification (Figure 1e′), a fine particle substrate is overlaid by branched fibers forming a hexagonal-like structure reminiscent of graphene. Literature attributes the increased porosity and surface roughness to high graphene oxide content and its uniform incorporation into the PLA matrix. These microstructural features are often linked to enhanced mechanical strength, thermal stability, and barrier properties in PLA/GO composites [47].

### 2.3. Hardness Test

**Figure 2:**
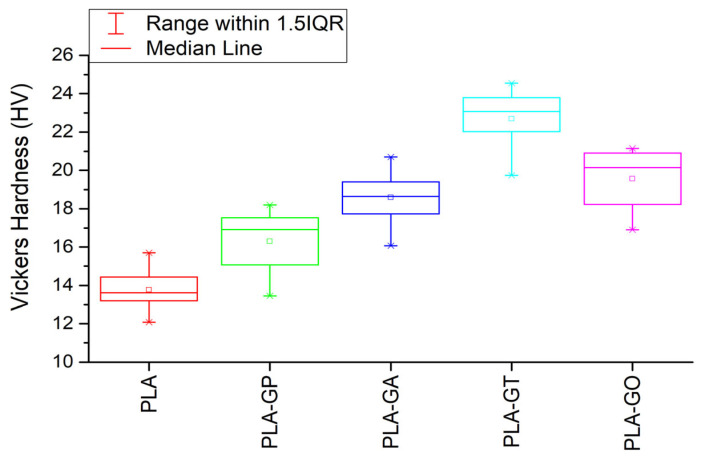
The results obtained of the hardness test and the standard deviation.

The hardness of PLA and its composites was evaluated using the Vickers test, and statistical analysis was performed via One-Way ANOVA (Figure 2). Median and mean hardness values increased progressively from neat PLA to PLA/0.5% GO–Ag, with slight overlap between the last two groups. Neat PLA exhibited the lowest hardness (13.77), whereas PLA/0.5% GO showed the highest values (19.56). The observed differences indicate a clear influence of the fillers on the material’s hardness.

Statistical analysis revealed a *p*-value of 0, confirming significant differences between groups at a 0.05 significance level. Tukey post hoc tests identified significant differences among all composites, except between PLA/0.5% GO and PLA/0.5% GO–Ag, for which differences were not statistically significant.

The addition of 0.5% grape pomace moderately increased hardness to 16.30, demonstrating reinforcement by natural fibers. Incorporation of 0.5% GO–Ag (18.59) and 0.5% GO (19.56) resulted in a more pronounced improvement, highlighting the stiffening effect of graphene oxide and its synergistic interaction with silver nanoparticles. The highest hardness was observed for PLA/0.5% GO–TiO_2_ (22.7), indicating strong interfacial interactions and efficient load transfer between the nanofillers and the PLA matrix. These results demonstrate that nanostructured fillers are more effective than natural fibers in enhancing the hardness of PLA composites, with GO–TiO_2_ providing the most substantial improvement. These findings indicate that nanostructured fillers are more effective than natural fillers in enhancing the hardness of PLA.

Overall, the combined results indicate that both natural and nanostructured fillers improve the mechanical performance, hardness, and microstructure of PLA-based films. Grape pomace provides moderate reinforcement through void formation and polymer–fiber interactions, whereas GO, TiO_2_, and silver nanoparticles contribute to enhanced stiffness, tensile strength, and hardness via strong interfacial bonding, efficient load transfer, and controlled pore formation. SEM analysis correlates with mechanical and hardness data, demonstrating that filler dispersion and surface morphology are critical determinants of composite performance. These results confirm that PLA-based nanocomposites, particularly those containing GO–TiO_2_, are promising candidates for durable and functional biodegradable food packaging.

### 2.4. The Antimicrobial Activity

**Figure 3:**
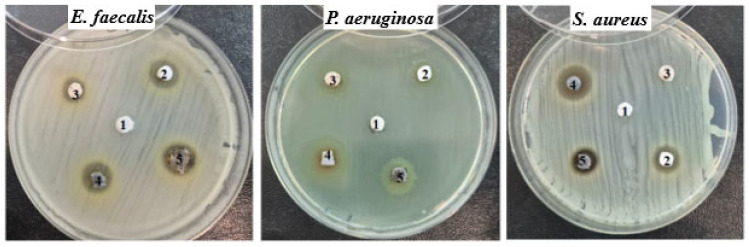
Antimicrobial effect of composite films on different bacterial strains: (1) PLA; (2) PLA-GP; (3) PLA-GA; (4) PLA-GT; (5) PLA-GO. Tested strains: *Enterococcus faecalis*, *Staphylococcus aureus,* and *Pseudomonas aeruginosa*.

The agar disc diffusion method revealed that all samples had a different inhibitory effect on both Gram-positive (*S. aureus* and *E. facecalis*) and Gram-negative bacteria (*P. aeruginosa*) are presented in Figure 3.

No inhibition was recorded in the control sample (1—PLA), and sample 3 (3—PLA-GA) also did not inhibit the *S. aureus* strain. The other tested samples (2—PLA-GP; 4—PLA-GT; 5—PLA-GO) showed inhibition zones between 10 and 15 mm.

**Figure 4:**
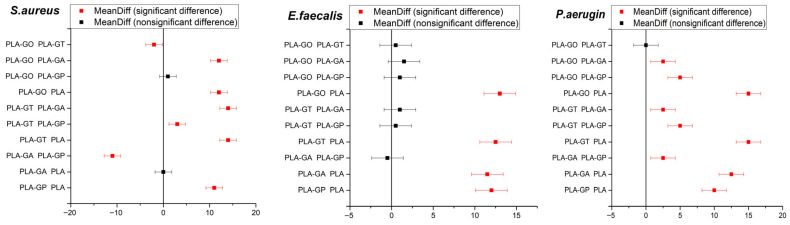
Tukey statistical analysis of antimicrobial activities.

One-way ANOVA statistical analysis was used to evaluate the differences in antibacterial activity among the PLA/Glycerol diacetate reference sample and PLA-based composites containing different nanoparticles against *S. aureus*, *E. faecalis*, and *P. aeruginosa*. The results presented in the Figure 4 revealed *p*-values lower than 0.05 for all three bacterial strains, indicating the presence of statistically significant differences among the analyzed groups. These findings confirm that modifying the PLA matrix by incorporating 0.5 wt. % functional fillers has a significant effect on the antibacterial behavior, irrespective of the bacterial strain tested.

The control sample based on PLA exhibited no inhibitory effect against *S. aureus*, *E. faecalis*, or *P. aeruginosa*. In contrast, all modified PLA samples demonstrated measurable antibacterial activity. Among them, PLA-GT and PLA-GO showed the highest inhibition zones, particularly against *P. aeruginosa* (15 mm), indicating strong antibacterial performance. PLA-GP exhibited moderate activity against all tested strains, while PLA-GA showed selective antibacterial behavior, being inactive against *S. aureus* but effective against *E. faecalis* and *P. aeruginosa*. These results suggest that surface or compositional modification of PLA significantly enhances its antibacterial properties, with the strongest effect observed for PLA-GT and PLA-GO.

Overall, the tested materials showed moderate antibacterial activity against Gram-positive strains, with inhibition zone diameters ranging from 11 to 14 mm for *E. faecalis* and *S. aureus*. This indicates that most of the samples possess some level of effectiveness against these bacteria, although the degree of inhibition varied depending on the sample composition.

Interestingly, a slightly higher antibacterial activity was observed against the Gram-negative bacterium *P. aeruginosa*. In particular, samples PLA-GA, PLA-GT, and PLA-GO exhibited notable inhibitory effects, with inhibition zones ranging from 12.5 to 15 mm. This enhanced activity against *P. aeruginosa* may be attributed to specific physicochemical properties of these samples, which could facilitate better interaction with or penetration through the bacterial cell envelope.

The observed differences in antibacterial performance among the samples suggest that variations in material composition or surface characteristics play a significant role in influencing antimicrobial efficacy. These findings highlight the potential of selected material formulations, especially samples PLA-GA, PLA-GT, and PLA-GO, as promising candidates for applications requiring antibacterial properties.

Overall, Gram-negative *P. aeruginosa* appears more susceptible to the modified PLA samples than *S. aureus*. Overall effectiveness (approximate ranking): PLA-GT ≈ PLA-GO > PLA-GP > PLA-GA >> PLA.

### 2.5. Evaluation of Thermal Characteristics by Differential Scanning Calorimetry (DSC)

**Figure 5:**
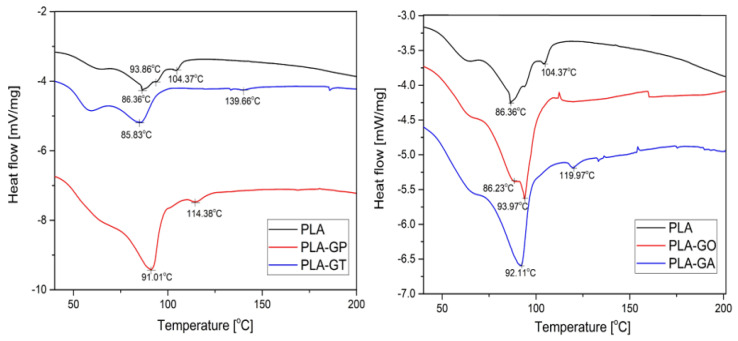
DSC curves of PLA composite gel (PLA) and their blends: PLA-GP; PLA-GT; PLA-GO, and PLA-GA.

The DSC curves of the investigated materials exhibited differences in the thermal behavior, according to Figure 5. All materials exhibited thermal transitions in the 53–59 °C range, attributed to the glass transition, indicating comparable segmental mobility of the amorphous phase.

The incorporation of inorganic nanoparticles (Ag and TiO_2_) shifted the endothermic processes toward higher temperatures, particularly for the PLA-GT sample, where transformations extended up to ~140 °C, suggesting increased thermal stability and structural rigidity. The PLA sample showed multiple successive endothermic effects characteristic of crystalline phase reorganization and melting, while the grape-pomace-based PLA-GP sample displayed intermediate behavior consistent with a heterogeneous structure.

The reference PLA sample exhibited an endothermic transition between 55.71 and 104.37 °C with a peak at 86.36 °C. The PLA-GO sample showed a transition between 56.84 and 93.97 °C with a peak at 86.23 °C. The PLA-GA composite presented a transition between 56.16 and 119.97 °C with a peak at 92.11 °C. The PLA-GT composite exhibited a transition between 59.36 and 139.66 °C with a peak at 85.83 °C. The PLA-GP composite showed a transition between 53.19 and 114.38 °C with a peak at 91.01 °C.

These results indicate that the incorporation of nanoparticles generally enhances the thermal stability and structural rigidity of the reinforced PLA composites, likely due to improved interfacial adhesion between the polymer matrix and the nanoparticles, which restricts polymer chain mobility. Nanoparticles can also act as nucleating agents, increasing crystallinity and modifying the glass transition and melting behavior. Variations in peak temperatures among the composites suggest that both the type and concentration of nanoparticles influence energy dispersion and the thermal degradation pathways of the material. This finding is consistent with previous studies [20,44]. For example, Balaji et al. reported that composites reinforced with titanium oxide and graphene oxide nanoparticles exhibited higher thermal stability, altered glass transition temperature, and improved viscoelastic properties [46].

## 3. Conclusions

The comprehensive characterization of the developed PLA-based composite gels demonstrates that the incorporation of both natural and nanostructured fillers significantly modifies and generally enhances the physicochemical performance of the polymer matrix.

Mechanical testing revealed that filler addition markedly improves tensile strength, stiffness, and hardness compared to neat PLA. Among the investigated systems, PLA containing 0.5% grape pomace exhibited the highest tensile strength and maximum load, while graphene oxide (GO)-based formulations, particularly GO–TiO_2_, showed substantial increases in elastic modulus and hardness. Statistical analysis confirmed that the observed differences among formulations were significant, highlighting the effectiveness of nanoparticle reinforcement compared to natural fillers.

SEM analysis confirmed significant morphological changes following filler incorporation. The presence of pores, layered structures, and well-dispersed nanoparticles correlated strongly with the observed mechanical and hardness properties. Uniform nanoparticle distribution and strong interfacial adhesion promoted improved load transfer and structural integrity, especially in nanocomposite systems.

Antimicrobial evaluation demonstrated that modified PLA samples exhibited measurable antibacterial activity against both Gram-positive and Gram-negative bacteria, whereas neat PLA showed no inhibitory effect. The strongest antibacterial performance was observed for PLA-GT and PLA-GO, particularly against *P. aeruginosa*, indicating the potential of nanostructured fillers to impart functional bioactivity to the polymer matrix.

Thermal analysis by DSC indicated that all formulations maintained similar glass transition temperatures, while inorganic nanoparticles improved thermal stability and influenced crystallization behavior. The decrease in cold crystallization temperature suggests that certain fillers act as nucleating agents, facilitating crystallization and modifying chain mobility without significantly altering melting temperature.

Overall, the results demonstrate that the incorporation of selected natural and nanostructured fillers effectively enhances the mechanical, thermal, structural, hardness, and antimicrobial properties of PLA. In particular, GO–TiO_2_–based composites show the most balanced and superior performance, making them promising candidates for advanced biodegradable food packaging applications where durability, functionality, and antimicrobial protection are required.

## 4. Materials and Methods

### 4.1. Materials

For the experimental investigations, five PLA-based gel systems were developed using polylactic acid (PLA; LUMINY^®^ LX975, Total Corbion PLA, Gorinchem, The Netherlands) as the primary polymer matrix. Their composition is shown in Table 2. Glycerol diacetate (Sigma-Aldrich Chemie GmbH, Taufkirchen, Germany) was employed as a plasticizing agent to promote polymer chain mobility and facilitate gel network formation. Structuring agents of inorganic origin—grape pomace, graphene oxide, and graphene oxide functionalized with silver or titanium dioxide nanoparticles—were incorporated to modulate the gel structure and functional performance. All structuring agents were synthesized by the Polymer Composites Laboratory of the Raluca Ripan Institute for Research in Chemistry [48,49].

Gel preparation was carried out via a solution-induced gelation approach. Briefly, 250 mL of chloroform was introduced into a 500 mL three-neck round-bottom flask equipped with a reflux condenser and a mechanical stirrer operating at 1500 rpm. The condenser enabled solvent reflux, ensuring constant system volume throughout processing. Under continuous stirring at ambient temperature, 84.3 g of PLA were gradually added to avoid polymer agglomeration and to promote uniform chain dispersion. Following complete polymer addition, the system temperature was raised to the solvent reflux point and maintained using an external thermostat until a homogeneous polymer solution was obtained.

Subsequently, 15 g of glycerol diacetate was incorporated to enhance chain flexibility and support the development of a viscoelastic gel network. After stabilization of the system, 0.5 wt% of the selected inorganic structuring agents was dispersed into the mixture. The resulting system was stirred continuously for 2.5 h to ensure uniform distribution and interaction between the polymer chains and the dispersed phases, leading to the formation of a structured gel state.

The obtained gel systems were then cast into petri dishes and allowed to undergo solvent evaporation–induced gel setting at room temperature. This process resulted in self-supporting gel films, stabilized through physical interactions within the polymer network. The incorporation of plasticizer and inorganic structuring agents was intended to tailor the mechanical flexibility, structural integrity, antimicrobial properties, and functional characteristics of the PLA-based gels, enhancing their suitability for food packaging applications.

**Figure 6:**
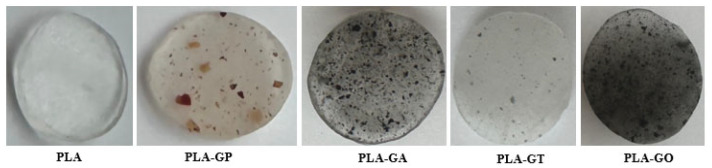
The PLA-based composite films were obtained after complete solvent evaporation.

Figure 6 presents the PLA-based formulations prepared after complete solvent evaporation. The reference sample consisted of a PLA/Glycerol diacetate blend containing 85 wt. % PLA and 15 wt. % Glycerol diacetate. In the modified systems, 0.5 wt. % of different fillers was incorporated into the PLA/Glycerol diacetate matrix, while the PLA content was reduced to 84.5 wt. % to maintain constant proportions. Specifically, grape pomace was added in Sample 2 (PLA-GP), silver-decorated graphene oxide (PLA-GA) in Sample 3, titanium dioxide-decorated graphene oxide (PLA-GT) in Sample 4, and graphene oxide (PLA-GO) in Sample 5.

### 4.2. Methods

#### 4.2.1. Mechanical Properties—Tensile Strength

The mechanical properties—tensile strength were evaluated using a Lloyd’s Instron Universal Analyzer, LR5K (Lloyd Instruments, Ameteklns, West Sussex, UK) at room temperature in accordance with UNE-EN ISO 527-3:2018. An axial load was progressively applied to each specimen (initial loading force: 5 N; maximum applied stress: 1000 N/mm^2^) until failure occurred. The specimens had a rectangular cross-section with dimensions of 3 mm in thickness, 4 mm in width, and 40 mm in length, featuring a calibrated central region and two clamping ends (3 × 25 × 25 mm) for secure fixation within the testing apparatus. From the resulting stress–strain curves, the elongation at fracture (ε), tensile strength (σ_u_), and Young’s modulus (E) were determined for each specimen. For result validation, each test group consisted of 30 specimens, with any deviation exceeding ±15% being excluded from the analysis. Statistical analyses were performed using one-way ANOVA in Origin 2019b. For each experimental group, the mean value was calculated from a minimum of 10 individual measurements. Post hoc comparisons between groups were conducted using Tukey’s test to identify statistically significant differences.

#### 4.2.2. SEM Microscopy

A Scanning Electron Microscopy (SEM) analysis was performed using an FEI Inspect S microscope (FEI Company, Hillsboro, OR, USA) operated in low vacuum mode with an acceleration voltage of 30 kV and a working distance (WD) of 8–12 mm. A secondary electron (SE) detector was used to capture high-resolution surface morphology, while the backscattered electron (BSE) detector was employed to highlight compositional contrast of the incorporated nanoparticles where necessary. Micrographs were acquired at different magnifications to evaluate the surface morphology and microstructural features of the PLA films.

#### 4.2.3. Hardness

Hardness tests were performed using the Duramin-40 AC3 apparatus (Struers GmbH, Fellbach, Germany).

Hardness measurements were performed using a Duramin-40 AC3 hardness tester (Struers GmbH, Fellbach, Germany), applying the Vickers method in accordance with ISO 6507. A test load of 100 gf was applied with a dwell time of 15 s. All tests were performed under controlled conditions to ensure reproducibility and accuracy. For each material, 10 measurements were taken, and the mean value was calculated.

#### 4.2.4. Antimicrobial Activity

The antimicrobial activity was evaluated on the fully dried films (5 mm in diameter and 1 mm in thickness) obtained after complete solvent evaporation, presented in Figure 6, using the agar disc diffusion method. Therefore, the reported antibacterial performance reflects the functional properties of the consolidated PLA-based composite films rather than the intermediate gel state.

The following microorganisms were used in this experiment: *Staphylococcus aureus* ATCC 25923, *Enterococcus faecalis* ATCC 29212, and *Pseudomonas aeuginosa* ATCC 27853. All bacteria used belong to the Microbiology Laboratory, Faculty of Biology and Geology from Babes Bolyai University. From each bacterial strain, a dilution of 0.5 McFarland in sterile saline water was made [50], which was spread over the entire surface of the Muller Hinton Agar medium. After that, a disk of each test sample (samples 2–5) and a control sample (sample 1). The bacterial strains were incubated at 25 °C for 2 days, and the diameter of the inhibition zone was measured. The diameter of the inhibition area is correlated with the sensitivity of the bacterium to the tested materials [51,52]. Each experiment was conducted three times, and the mean was calculated.

#### 4.2.5. Evaluation of Thermal Characteristics by Differential Scanning Calorimetry (DSC)

Thermal analyses were performed by differential scanning calorimetry (DSC) using a DSC 630e calorimeter (Mettler-Toledo, Singapore) with a maximum operating temperature of 750 °C. Measurements were carried out under a nitrogen atmosphere in a 40 μL aluminum crucible at a heating rate of 10 °C/min over the temperature range of 25–250 °C. An isothermal hold of 0.5 min was applied at the final temperature. The nitrogen flow rate was maintained at 80 mL/min. The glass transition temperature (Tg), crystallization temperature (Tc), and melting temperature (Tm) were determined from the recorded thermograms.

## Figures and Tables

**Table 1:** Mechanical properties of PLA and its composite gels (Mean ± Standard Deviation).

Experimental Materials	Tensile Strength (MPa)	Maximum Load (N)	Elongation at Fracture (mm)	Young’s Modulus (MPa)
PLA	1.48 ± 0.2 ^a^	36.33 ± 2.8 ^a^	180.71 ± 2.2 ^a^	2.87 ± 1.2 ^a^
PLA-GP	7.38 ± 0.6 ^b^	189.32 ± 15.5 ^b^	66.71 ± 1.5 ^b^	105.12 ± 15.4 ^b^
PLA-GA	4.40 ± 0.5 ^c^	138.76 ± 14.7 ^c^	50.87 ± 2.5 ^b^	57.02 ± 9.3 ^c^
PLA-GT	2.13 ± 0.1 ^a^	35.99 ± 3.6 ^a^	248.44 ± 4.5 ^c^	2.60 ± 0.8 ^a^
PLA-GO	1.81 ± 0.2 ^a^	75.72 ± 16.4 ^d^	1.58 ± 0.2 ^d^	102.38 ± 19.9 ^b^
*p*	<0.05			

Groups sharing the same superscript letter are not significantly different according to Tukey’s post-hoc test. Different letters indicate statistically significant differences (*p* < 0.05).

**Table 2:** The composition of experimental PLA composite gels.

Experimental Materials	Composition wt. %	Filler 0.5 wt. %
PLA	Polylactic acid (PLA) Glycerol diacetate (15%)	-
PLA-GP		Grape pomace
PLA-GA		GO-Ag
PLA-GT		GO-TiO_2_
PLA-GO		GO

## Data Availability

The original contributions presented in this study are included in the article; further inquiries can be directed to the corresponding authors.
